# α-Tocopherol Attenuates Oxidative Phosphorylation of CD34^+^ Cells, Enhances Their G0 Phase Fraction and Promotes Hematopoietic Stem and Primitive Progenitor Cell Maintenance

**DOI:** 10.3390/biom11040558

**Published:** 2021-04-10

**Authors:** Laura Rodriguez, Pascale Duchez, Nicolas Touya, Christelle Debeissat, Amélie V. Guitart, Jean-Max Pasquet, Marija Vlaski-Lafarge, Philippe Brunet de la Grange, Zoran Ivanovic

**Affiliations:** 1 Etablissement Français du Sang Nouvelle Aquitaine, Place Amélie Raba Léon, CS22010, CEDEX, 33075 Bordeaux, France; laura.rodriguez@efs.sante.fr (L.R.); pascale.duchez@efs.sante.fr (P.D.); nicolas.touya@u-bordeaux.fr (N.T.); Marija.Vlaski@efs.sante.fr (M.V.-L.); Philippe.Brunet-De-La-Grange@efs.sante.fr (P.B.d.l.G.); 2 Inserm Bordeaux UMR 1035, 33000 Bordeaux, France; christelle.debeissat@u-bordeaux.fr (C.D.); amelie.guitart@u-bordeaux.fr (A.V.G.); jean-max.pasquet@u-bordeaux.fr (J.-M.P.); 3 Université de Bordeaux, 33000 Bordeaux, France

**Keywords:** α-tocopherol acetate, hematopoietic stem cells, hematopoietic progenitors, electron transport chain, proliferative capacity, quiescence, energetic metabolism, oxidative phosphorylation

## Abstract

Alpha tocopherol acetate (αTOA) is an analogue of alpha tocopherol (αTOC) that exists in the form of an injectable drug. In the context of the metabolic hypothesis of stem cells, we studied the impact of αTOA on the metabolic energetic profile and functional properties of hematopoietic stem and progenitor cells. In ex vivo experiments performed on cord blood CD34^+^ cells, we found that αTOA effectively attenuates oxidative phosphorylation without affecting the glycolysis rate. This effect concerns complex I and complex II of the mitochondrial respiratory chain and is related to the relatively late increase (3 days) in ROS (Reactive Oxygen Species). The most interesting effect was the inhibition of Hypoxia-Inducible Factor (HIF)-2α (Hexpression, which is a determinant of the most pronounced biological effect—the accumulation of CD34^+^ cells in the G0 phase of the cell cycle. In parallel, better maintenance of the primitive stem cell activity was revealed by the expansion seen in secondary cultures (higher production of colony forming cells (CFC) and Severe Combined Immunodeficiency-mice (scid)-repopulating cells (SRC)). While the presence of αTOA enhanced the maintenance of Hematopoietic Stem Cells (HSC) and contained their proliferation ex vivo, whether it could play the same role in vivo remained unknown. Creating αTOC deficiency via a vitamin E-free diet in mice, we found an accelerated proliferation of CFC and an expanded compartment of LSK (lineage^negative^ Sca-1^+^cKit^+^) and SLAM (cells expressing Signaling Lymphocytic Activation Molecule family receptors) bone marrow cell populations whose in vivo repopulating capacity was decreased. These in vivo data are in favor of our hypothesis that αTOC may have a physiological role in the maintenance of stem cells. Taking into account that αTOC also exhibits an effect on proliferative capacity, it may also be relevant for the ex vivo manipulation of hematopoietic stem cells. For this purpose, low non-toxic doses of αTOA should be used.

## 1. Introduction

The parenteral form of a therapeutic agent for the treatment of vitamin E deficiency is alpha tocopherol acetate (αTOA). In vivo αTOA is metabolized by cells, and the acetyl group is removed by an esterase to generate the active form of alpha-tocopherol (αTOC) [1,2]. As an injectable drug, this molecule is usable in ex vivo cultures and is very appropriate for clinical grade cell engineering. In our previous work with mesenchymal stromal cells [3], we discovered an interesting action of αTOA ex vivo: it decreases the mitochondrial oxygen consumption rate (OCR) and increases mitochondrial reactive oxygen species ROS without affecting glycolysis. Furthermore, αTOA in parallel enhances maintenance of the proliferative capacity and primitiveness of multipotent mesenchymal stem cells without affecting proliferation itself. This feature is in line with our metabolic hypothesis [4,5], as well as with observations of other authors [6,7], that low oxidative phosphorylation (OXPHOS) activity is in direct correlation with a higher level of stemness of hematopoietic stem cells (HSC) and vice versa. On the other hand, the mechanism of this relationship is not clear. In fact, each cellular type might exhibit a different way of regulating the level of stemness in respect of its metabolic energetic profile.

When αTOA, as an injectable medical agent, was used for creation of the cell preservation medium in the context of the preservation of hematopoietic progenitor cells (HPC) in hypothermia (https://patents.google.com/patent/FR3040860A1/fr (EP 167234430.1 delivered 24 June 2020, N° 3285576)), its effect of decreasing OCR was also observed. This finding inspired us to further explore the influence of αTOA on the energetic metabolic profile of cord blood CD34^+^ cells in normothermia.

Here, we present the results of our pioneering study concerning the effect of αTOA on the energetic metabolism and functional properties of hematopoietic stem and progenitor cells (HSPCs) ex vivo, as well as the effect of αTOA on these cell populations in vivo. We confirm the effect of αTOA on the attenuation of mitochondrial respiration and its action in favor of the maintenance of the quiescent cell fraction, which appears to be related to the inhibition of the Hypoxia-Inducible Factor (HIF)2-α expression. We also show the effect of αTOA on the maintenance of the proliferative capacity of primitive progenitors and stem cells.

Acquired ex vivo data are compatible with the in vivo data showing an opposite effect on HSPC proliferation in the state of induced αTOC deficiency. Thus, we conclude that αTOC may be a relevant agent for cell engineering since it can provide a way to control the quiescence and proliferative capacity of HSPCs.

## 2. Materials and Methods

### 2.1. Cord Blood CD34^+^ Cells

CD34^+^ cells were isolated by Miltenyi’s indirect immunomagnetic procedure (Magnetic-activated cell sorting (MACS) human CD34^+^ progenitor cell isolation kit, Miltenyi Biotec, Paris, France) with magnetic selection MS columns (Vario MACS device, Miltenyi Biotec, Paris, France) from the cord blood (CB) units noted as “not suitable for banking” at the CB Bank of Bordeaux, French Blood Institute. After selection, CD34^+^ cells were either used directly for the experiments or were frozen for later use [8].

### 2.2. CD34^+^ Expansion Model

CD34^+^ cells from CB (2 × 10^5^ cells/mL) were seeded with different concentrations of αTOA (0 µM, 20 µM, 40 µM, and 150 µM) in the culture medium supplemented with cytokines from Peprotech (Neuilly-Sur-Seine, France): SCF (Stem Cell Factor), (FMS-Like Tyrosine Kinase 3 receptor Ligand) Flt3L (100 ng/mL each) G-CSF (10 ng/mL), and TPO (20 ng/mL). The αTOA doses (20 µM and 40 µM) were chosen on the basis of plasma αTOC concentrations in human serum [9,10]. To comprehensively cover the αTOC effect, we also used a higher dose (150 µM) in order to evaluate αTOC toxicity. Owing to the difficulties in procuring the same medium throughout the study, a set of different media was used for all analyses (HP01 medium, Macopharma, Tourcoing, France, HPGM and X-VIVO 15 medium, Lonza, Basel, Switzerland) and the same effect of αTOA was observed with all the different media used.

In order to analyze energetic metabolism (oxygen consumption rate, glycolytic activity, ATP and ROS level) in the first step of expansion, CD34^+^ cells were cultured in the above-described conditions for only 24 h at 37 °C + 5% CO_2_ before analysis. For the other assays, CD34^+^ cells were cultured for 3 days in the same conditions prior to the analysis of the secondary culture.

After 3 days of incubation of the primary culture, cells were washed to remove αTOA and the secondary culture (2 × 10^4^ cells/mL) was started in the same medium supplemented with the same cytokines. The primary/secondary culture approach is used to detect the activity of the primitive progenitors preceding colony forming cells (pre-CFC) [11]. We chose to compare different time-points in the kinetics of the expansion at the beginning and during the secondary culture: day 3, day 7, day 10, day 14, day 17, etc.

### 2.3. Colony-Forming Units Assay

For each analysis at day 0 (start of the primary culture), day 3 (end of the primary and start of the secondary culture), and for different time-points during the secondary culture, 50 cells were plated in cytokine-supplemented methylcellulose (H4034, stem cell technology, Grenoble, France) in 24-well plates in duplicate. After 14 days of incubation at 37 °C in a water-saturated atmosphere (20% O_2_ and 5% CO_2_), the colony-forming units (CFUs) were counted using an inverted microscope (Nikon, Champigny sur Marne, France).

### 2.4. Viability and Apoptosis Assay

In order to investigate cell viability after 3 days of primary culture with αTOA at different concentrations, we used an Annexin V/propidium iodide (PI) kit (Beckman Coulter, Villepinte, France) paired with CD34^+^ immunolabelling (anti-human CD34-APC, Beckton Dickinson, Pont de Claix, France). Cells were analyzed with a BD FACS Canto II flow cytometer (Becton Dickinson, Rungis, France), after which we were able to evaluate 3 sub-populations: viable cells (Ann^−^, PI^−^), early apoptotic cells (Ann^+^, PI^−^) and apoptotic/necrotic cells (Ann^+^, PI^+^).

### 2.5. Cell Cycle Analysis

To analyze the quiescent state (G0 phase), G1 phase, or proliferative phases (S, G2, M) of the CD34^+^ cells after 3 days of primary culture with αTOA, we used Ki67/PI labelling after fixation and permeabilization steps. Cells were incubated overnight at 4 °C in phosphate-buffered saline (PBS) + 0.5% formaldehyde, then washed in PBS and incubated for a minimum of 2 h in 80% ethanol at −20 °C. Cells were washed in PBS and labelled with Alexa Fluor 647 anti-human Ki67 antibody (Beckton Dickinson, Pont de Claix, France) in a 0.25% Triton X-100 buffer for 1 h at 4 °C in the dark, then washed and resuspended in PBS–ribonuclease (1 mg/mL)–PI (50 mg/mL) buffer prior to analysis on a BD FACS Canto II flow cytometer.

In parallel, fixed and permeabilized cells were labelled with FITC anti-human cyclin D1 antibody set (#554109 BD Biosciences) for 1 h at 4 °C in the dark, then washed and resuspended in PBS prior to analysis on a BD FACS Canto II flow cytometer.

For the tracking of cell division, fresh CD34^+^ cells at 10 × 10^6^ /mL were incubated on day 0 with 5 µM Carboxyfluorescein succinimidyl ester (CFSE) dye (#C34570, Thermo Fisher Scientific, Bordeaux, France) for 15 min at 37 °C and then washed twice with warm PBS. After 3 days of primary culture, cells were separated into two populations with a cell sorter (FACS ARIA, Becton Dickinson, Rungis, France) according to their fluorescence intensity. The CFSE^high^ population (same fluorescence intensity as day 0), represents cells which did not undergo division, while CFSE^low^ cells represent the population which had divided at least once. All cell populations were fixed and permeabilized for Ki67/PI labelling as described above.

### 2.6. Analysis of Primitive HSC Activity (Pre-SRCs)

CD34^+^ cells treated with or without αTOA. After 3 days of primary expansion, the culture (PEC) was intravenously injected into 12-week-old NOD scid gamma mouse (NSG) mice (one part of the PEC), or washed and seeded in a secondary culture for 10 days (second part of the PEC), and then injected into a second group of mice. On day 0, 1000 cells were injected per mouse, or a number of cells representing their progeny (equivalent) were injected on day 3. On day 10, the equivalent of 1/10 of the day 0 cell progeny was injected. This reduction in the injected cell proportion was necessary in order to avoid saturation (high plateau chimerism) in the recipient mice, which can occur due to the expansion of reconstituting HSCs and would prevent the adequate comparison between 0 µM and 20 µM αTOA treatments.

Eight weeks after injection, bone marrow from the femurs of recipient mice was collected, counted and analyzed by flow cytometry (BD FACS Canto II, Rungis, France) for human CD45 chimerism (FITC anti-human CD45, #A07782, Beckman Coulter, Villepinte, France).

### 2.7. Analysis of Mitochondrial ROS (MitoSOX)

The level of mitochondrial ROS was quantified by the fluorescent probe MitoSOX (Thermo Fisher Scientific, M366008) in CD34^+^ cells treated with or without αTOC after 3 days of primary culture. To measure ROS produced by the mitochondria, the cells were washed and resuspended in Hanks’ Balanced Salt Solution (HBSS) (Thermo Fisher Scientific #14025092); then, 5 μM of MitoSOX was added and the cells were incubated for 15 min in the dark at 37 °C. Next, 50 µM verapamil was added in order to block the efflux pumps that reject the probe. After the incubations, cells were washed twice with PBS pre-warmed to 37 °C (Lonza, #17-516F, Basel, Switzerland). Intracellular fluorescence was detected by flow cytometry on a BD FACS Canto II and analyzed using FACSDiva software (Becton Dickinson, Rungis, France).

### 2.8. Metabolic Analysis

The measurement of energetic metabolism of CD34^+^ cells in each condition (with or without αTOC) was performed with Seahorse XF extracellular flux analyzers (Agilent technology, Santa Clara, CA, USA). We performed a mitochondrial stress test, glycolysis stress test and analysis of individual activity of complex I, complex II and complex IV. Before performing these tests, cells were glued in a reversible way with Cell-Tak (7 µg/cm^2^, Corning Life Science, Corning, NY, USA) and seeded just before analysis in the Seahorse plate at 2 × 10^5^ cells per well in triplicate for each condition. Measurements were recorded for 133 min and analyzed using Microsoft Excel.

### 2.9. Mitochondrial Stress Test

The mitochondrial stress test allows us to determine the basal respiration, ATP production, spare capacity, maximal respiration and non-mitochondrial oxygen consumption of CD34^+^ cells treated with or without αTOA. The cells were washed with PBS and the medium was replaced with pre-warmed XF Base Medium (part# 102353-100) supplemented with 100 mM pyruvate (Sigma Aldrich, St Louis, MO, USA, #P8574), 200 g/L glucose (Sigma Aldrich, #G8270) and 200 mM ultra-glutamine (Lonza, Basel, Switzerland, #BE04-684E), pH 7.4. The oxygen consumption rate (OCR) was recorded in real time before and after the subsequent injections of the modulatory compounds A, B, and C (A: 1.27 μM oligomycin (an ATP synthase inhibitor; #75351, Sigma Aldrich, USA), B: 100 μM of 2,4 Dinitrophenol (DNP) (a decoupling agent; #D198501 Sigma Aldrich, USA), and C: a mix of 1 μM rotenone (#R8875, Sigma Aldrich, USA) and 1 μM antimycin (#A8674 Sigma Aldrich, USA), which are inhibitors of complex I and complex III of the electron transport chain, respectively).

### 2.10. ATP Quantification by Luminescence

In order to correctly interpret ATP production, which was indirectly calculated from the Seahorse data, we chose to quantify ATP by a more sensitive method using the CellTiter-Glo^®^ 2.0 Assay (G9241, Promega, Madison, WI, USA). This test provides a homogeneous method to determine the number of viable cells in culture by quantifying the amount of ATP present, which indicates the presence of metabolically active cells. We used ATP disodium salt (Cat.# P1132, Sigma Aldrich, Saint Louis, MO, USA) to generate the standard curve. The amount of ATP was determined after 24 h of culture on exactly 2000 CD34^+^ cells for all concentrations of αTOA tested (0 µM–150 µM).

### 2.11. Glycolysis Stress Test

The glycolysis stress test, performed using Seahorse technology, allowed us to determine the basal glycolysis level, maximum/spare glycolytic capacity and non-glycolytic acidification. Cells were washed with PBS and the medium was replaced with pre-warmed XF Base Medium (part# 102353-100), pH 7.4. The extracellular acidification rate (ECAR) was recorded in real time before and after subsequent injections of the modulatory compounds A, B and C (A: 2 g/L glucose (#G8270, Sigma Aldrich, Saint Louis, MO), B: 1.27 μM oligomycin (#75351, Sigma Aldrich, Saint Louis, MO, USA), C: 100 mM 2–deoxy glucose, pH 7.4 (#D6134, Sigma Aldrich, Saint Louis, MO, USA)).

### 2.12. Analysis of Global Mitochondrial Activity

Global mitochondrial activity was quantified using the fluorescent probe tetramethylrhodamine methyl ester (TMRM; #M20036, Thermo Fisher Scientific, Waltham, MA, USA) in CD34^+^ cells treated with or without αTOA after 3 days of primary culture. The mitochondrial membrane potential state was determined directly by flow cytometry on the basis of a red–orange fluorescent signal emitted by TMRM (the signal diminishes with decreases in membrane potential). TMRM was added to 2 × 10^5^ CD34^+^ cells (final concentration: 20 nM) treated with or without αTOA after 3 days in primary culture and incubated for 30 min in the dark at 37 °C. Then, 50 µM verapamil was added to each condition in order to block the efflux pumps that reject the probe. After the completed incubations, the cells were washed with cold PBS. Fluorescence was detected by a BD FACS Canto II flow cytometer using FACSDiva software. In further steps, cells were incubated with 100 µM DNP (2,4-dinitrophénol; Sigma Aldrich, St Louis, MO, USA) for 10 more minutes in the dark at 37 °C, washed, and the fluorescence was recorded for a second time. The difference between the levels of fluorescence before and after DNP was added corresponds to the specific signal of mitochondrial activity.

### 2.13. Individual Activity of Complex I, Complex II and Complex IV

To investigate how αTOA impacts the respiratory chain, we chose to apply the method described by Salabei et al. [12] using the Seahorse device (Agilent Technologies, Santa Clara, CA, US). We individually analyzed the activity of respiratory chain complexes I, II and IV, as well as the rate of substrate use by each complex in permeabilized CD34^+^ cells treated with or without αTOA for 24 h. On the day of the experiment, CD34^+^ cells were washed with PBS and the culture medium was replaced with mannitol and sucrose buffer (MAS) pre-warmed at 37 °C, containing 70 mM sucrose (#573113, Calbiochim Sigma Aldrich), 220 mM mannitol (#A14030 Alfa Aesar by Thermo Fisher), 10 mM KH_2_PO_4_ (#529568, Calbiochim Sigma Aldrich), 5 mM MgCl_2_ (#M8266, Sigma Aldrich), 2 mM HEPES (4-(2-hydroxyethyl)-1-piperazineethanesulfonic acid #A14777 Alfa Aesar by Thermo Fisher) and 1 mM EGTA (Ethylene glycol-bis(2-aminoethylether)-N,N,N′,N′-tetraacetic acid; E4378, Sigma Aldrich), pH 7.2. In this Seahorse protocol, we measured the oxygen consumption rate (OCR) of permeabilized cells treated with 25 μg/mL digitonin (# D141, Sigma Aldrich) in real time. First, we injected the specific substrate of the analyzed complex (2 mM malate (#M0875, Sigma Aldrich) for complex I, 10 mM succinate (#S3674, Sigma Aldrich) for complex II, and ascorbate (#A 4544, Sigma Aldrich) for complex IV). Next followed the injection of 1.27 μM olygomycin (#75351, Sigma Aldrich) and finally the injections of the specific inhibitors for each tested complex (10 μM rotenone (#R8875, Sigma Aldrich) for complex I, 10 μM antimycin (#A8674, Sigma Aldrich) for complex II, and Azide K (#740411, Sigma Aldrich) for complex IV) [12].

### 2.14. Western Blot of HIF-α Proteins

Proteins were extracted from CD34^+^ cells exposed to 0 μM, 20 μM and 40 μM of αTOA for 72 h at 20% O_2_ using cell lysis buffer (#9803, Cell Signaling Technology, Danvers, MA, USA). For the positive control, CD34^+^ cells were exposed to 100 μM cobalt chloride solution (CoCl_2_; #15862, Sigma Aldrich) or hypoxic conditions (0.1% O_2_) for 6 h in the same culture conditions as above, and total proteins were obtained using the same lysis buffer. For the negative control, mononucleated cells (MNCs) from CB were extracted on day 0 and total proteins were obtained using the same lysis buffer. Precision Plus Protein™ Dual Color Standards (#1610374, BioRad, Hercules, CA, USA) were used as protein markers.

Western blot analyses were performed with 30 µg of the total protein extract per condition (with/without αTOA). The protein extracts were separated according to molecular weight using ready-to-use 8–16% Mini-PROTEAN^®^ TGX™ Precast Protein Gels (#4561103, BioRad, Hercules, CA, USA) in a BioRad electrophoresis system (power at 25 mA (per gel) for 90 min).

The separated proteins were transferred onto Hydrophilic polyvinylidene fluoride (PVDF) membrane (#1620177, BioRad, Hercules, CA, USA) with a wet transfer system for 2 h at 300 mA with FlashBlot transfer buffer (R-03090-D50, Advansta, San Jose, CA, USA). The membranes were blocked with Tris buffered saline with 0.1% Tween-20 (TBS–T) supplemented with 5% fat dried milk for 1 h at room temperature under agitation. After saturation, the membranes were incubated with 1:1000 HIF-1α (Abcam, #ab51608, Cambridge, UK), 1:500 HIF-2α (#NB100-132, Novus Biologicals, Bio-Techne, Centennial, CO, USA), or with 1: 5000 β-actin (#8226, Abcam, Cambridge, UK) primary antibody overnight at 4 °C under soft agitation. The blots were washed 3 times with 1× TBS-T at room temperature and then incubated with horseradish peroxidase-conjugated rabbit (R-05072-500, Advansta, San Jose, CA, USA) or mouse (R-05071-500, Advansta, San Jose, CA, USA) secondary antibody for 1 h at room temperature. Then, the membranes were washed 3 times and the immunoreactive proteins were detected by using the WesternBright Sirius Chemiluminescent Detection Kit (#K-12043-D10, Advansta, San Jose, CA, USA) and a Fusion Solo S6 edge device (Vilber Lourmat, France). Quantum software was used for the band intensity analysis (Vilber Lourmat, France).

### 2.15. qPCR

At the same time as protein extraction, some CD34^+^ cells were used to isolate total RNA using TRIzol reagent (#15596026 Invitrogen, Villebon-sur-Yvette, France), and reverse transcription was performed using a QuantiNova™ Reverse Transcription Kit (#205410, Quiagen, Courtaboeuf, France). The purity and quantity of the extracted RNA were verified with a Nanodrop device (Thermo Fisher Scientific, Waltham, MA, USA). qPCR was performed using Master Mix Applied Biosystems™ PowerUp™ SYBR™ Green (#A25780, Thermo Fisher Scientific) in a 384-well plate thermocycler (CFX 384 Touch PCR real-time detection system Bio-Rad, Hercules, CA, USA). The sequences of the forward/reverse *HIF*-*α* primers used are GCCCAATAGCCCTGAAGACT/TGAAATCCGTCTGGGTACTGC, which were validated with good efficacy (>95%).

### 2.16. Transduction Model of shHIF-α

In order to better understand the effect of αTOA on HIF-2α, we induced *HIF-1α* and *HIF-2α* shutdown via the lentiviral transduction of CD34^+^ cells. All lentivirus vectors were produced using the vectorology platform Vect’UB (INSERM US 005—CNRS 3427—TBMCore, Bordeaux University, France). CD34^+^ cells were transduced for 3 days with small hairpin RNA against HIF-1α (shHIF-1α) and/or against HIF-2α (shHIF-2α) for each experimental condition, and with shCTRL for the control group in Iscove Modified Dulbecco Media (IMDM) (Gibco), 50% BIT9500 (StemCell Technologies, Saint-Egrève, France) and the cytokines (Peprotech) SCF (100 ng/mL), FLT3-L (100 ng/mL), IL-6 (60 ng/mL) and TPO (20 ng/mL). Cells were seeded at a concentration of 1 × 10^6^ cell/mL and transduced at 50 Multiplicity of infection (MOI) (25 MOI added on day 1 and 25 MOI added on day 2). After 3 days of transduction, cells were washed twice with PBS and GFP positive cells were sorted (BD FACS Aria, Beckton Dickinson, Rungis, France) to select only the transduced cells. GFP negative cells were kept for controls as non-transduced cells.

### 2.17. Murine αTOC Deficiency Model

These experiments were performed after approval from the Bordeaux Ethics Committee for animal experimentation (French National Ministry of High Education and Research authorization (APAFIS#13468-2018020914295792v4)). Three-week-old C57BL/6 CD45.2 mice (Charles River, Écully, France) were divided into 2 groups: (1) mice receiving vitamin E-free food (custom food U8959, Safe nutrition, Augy, France) (“deficiency” group), and (2) mice receiving the same U8959 food supplemented with 40 mg/kg of αTOA (“control” group). αTOC deficiency was validated after 2 months by quantification of the αTOC levels in mouse serum using HPLC (subcontracted by Eurofins Biomnis, Ivry-sur-Seine, France). After 2 months, the mice were sacrificed and the total number of cells, CFC, LSK and SLAM compartments were determined in their femoral bone marrow. The LSK and SLAM compartment were evaluated by flow cytometry. Bone marrow cells were incubated with PE anti-mouse CD3 (#100205), PE anti-mouse CD4 (#100407), PE anti-mouse CD8a (#100707), PE anti-mouse CD11b (#101207), PE anti-mouse B220 (#103207), PE anti-mouse Ly-6G (#108407), PE anti-mouse Ter119 (#116207), FITC anti-mouse CD117 (#105805) and APC anti-mouse Sca1 (#108111) antibodies for LSK determination, and BV421 anti-mouse CD48 (#103427) and BV510 anti-mouse CD150 (#115929) antibodies for SLAM determination. All antibodies were provided by BioLegend. S phase quantification was determined by the Ara-C suicide kit (cytosine-arabinoside; Cytarabine, #C3350000, Merck, Darmstadt, Germany). Femoral bone marrow (BM) cells were incubated for 1 h at 37 °C with or without Ara-C (50 µg/mL) and washed twice before being seeded (20,000 cells) in 1 mL of methylcellulose (R&D system, HSC007, Minneapolis, MN) and placed in a 35 mm petri dish in duplicate. After 7 days, the colonies were counted in both conditions. The percentage of reduction in the colony number in the Ara-C treated condition corresponds to the percentage of HPCs in the S phase of the cell cycle. To test their engraftment capacity, 100,000 cells from each donor mouse (5 mice in each group) were injected into a single C57BL/6 CD45.1 recipient mouse (i.v.) (Charles River). Four months after injection, recipient mice were sacrificed and their bone marrow cells were extracted and incubated with Alexa Fluor 700 anti-mouse CD45.1 (#110723, BioLegend), PE/Cy7 anti-mouse CD45.2 (#109829, BioLegend) and LSK-SLAM antibodies as described above. Cells were analyzed by flow cytometry (BD FACS Canto II) to determine the percentage of CD45.2 donor cells and to evaluate the LSK-SLAM compartment.

### 2.18. Statistical Analysis

Data from each cohort are presented as mean +/− standard deviation. The non-parametric Mann–Whitney test was used to compare experimental conditions for both in vitro and in vivo assays when two cohorts were compared. For the comparison of multiple cohorts, the Kruskal–Wallis test followed by the Mann–Whitney test with Bonferroni correction was used.

## 3. Results

### 3.1. α-TOA Inhibits the Amplification of Committed Progenitors in Culture and Induces the Accumulation of CD34^+^ Cells in G0 Phase

**Figure 1:**
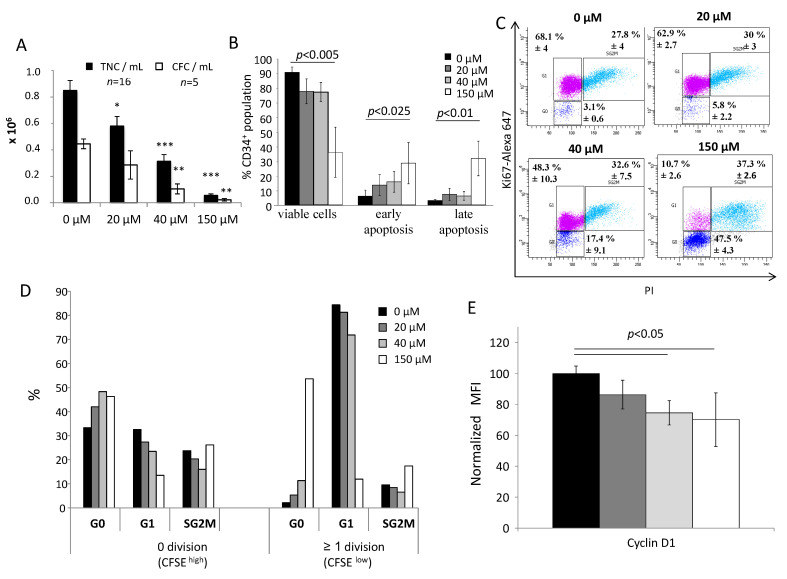
Influence of αTOA on proliferative kinetics and the cell cycle of CD34^+^ and committed progenitor cells. (**A**) αTOA inhibits the amplification of total nucleated cells (TNC) and committed progenitors (CFC) after 3 days of primary culture. *: *p* < 0.025, **: *p* < 0.01 and ***: *p* < 0.001 in comparison to the 0 µM condition. (**B**) Cell viability of CD34^+^ cells after 3 days of primary culture, *n* = 6. (**C**) Cell cycle labelling shows an accumulation of CD34^+^ cells in the G0 phase after 3 days of αTOA treatment, *n* = 6. (**D**) The G0 accumulation increases with αTOA concentration. *n* = 2. (**E**) αTOA inhibits cyclin D1 after 3 days of culture *n* = 5. Data presented in (**A**,**B**,**C**,**E**) are means +/− SD.

After 3 days of CD34^+^ cell culture in the presence of different doses of αTOA, an inhibitory effect on the proliferation of the cultured cells was detected when compared to the culture without αTOA (Figure 1A). The same phenomenon was found for the committed progenitors (CFC) (Figure 1A). It should be stressed that the 150 µM dose almost completely suppressed the CFCs. This coincided with a drastic increase in apoptotic cells and decrease in cell viability (Figure 1B), as well as with the suppression of SRC activity (Figure S1). αTOA did not significantly affect cell viability in culture when the αTOA concentration was within the physiological range. However, αTOA induced apoptosis and decreased the number of viable CD34^+^ cells when it was applied in supra-physiological doses (150 µM) (Figure 1B). In the bulk cell population, after 3 days of culture, αTOA increased the proportion of cells in the G0 phase of the cell cycle at the expense of G1 phase cell proportion in a dose-dependent manner (Figure 1C). This phenomenon concerns mainly the cells which did not divide, and to a lesser extent, the cells that performed at least one cellular division (Figure 1D). This phenomenon of the increased number of cells in the G0 phase is regulated via decreased cyclin D1 expression in correlation with αTOA concentration (Figure 1E).

### 3.2. The Potential for Generating Total Nucleated Cells and Committed Progenitors in Secondary Cultures Is Higher When αTOA Is Applied in Primary Cultures

**Figure 2:**
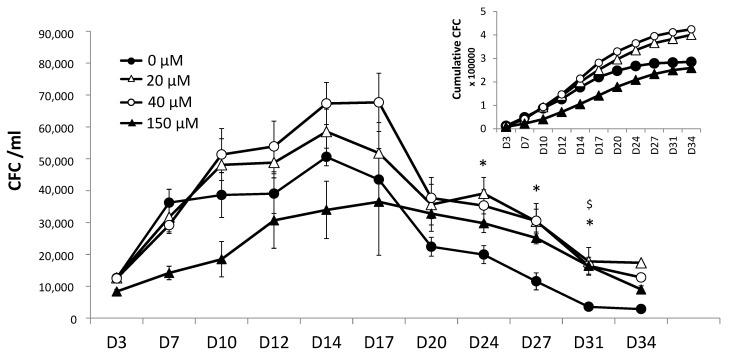
Potential for generating committed progenitors in secondary culture, *n* = 6. The cumulative curve, where the value for each time point is a sum of the values for all previous time points, is presented in the inset. αTOA was present only in the primary culture (for 3 days); the cells were then washed and seeded in the same supplemented medium in the secondary cultures. Data are presented as mean +/− SD. * *p* < 0.05 (0 µM compared to 20µM), $ *p* < 0.05 (0 µM compared to 40 µM).

When the primary culture had been supplemented with physiologically relevant doses (20 and 40 µM) of αTOA for 3 days, we subsequently detected a higher production of CFC in the secondary culture (Figure 2). This effect is clearly evident from the cumulative curve (inset). The supra-physiological dose of 150 µM αTOA led to a decrease in CFC production during the secondary culture vs. the control condition.

### 3.3. αTOA Preserves a Fraction of the Primitive Repopulating Cells in Culture

**Figure 3:**
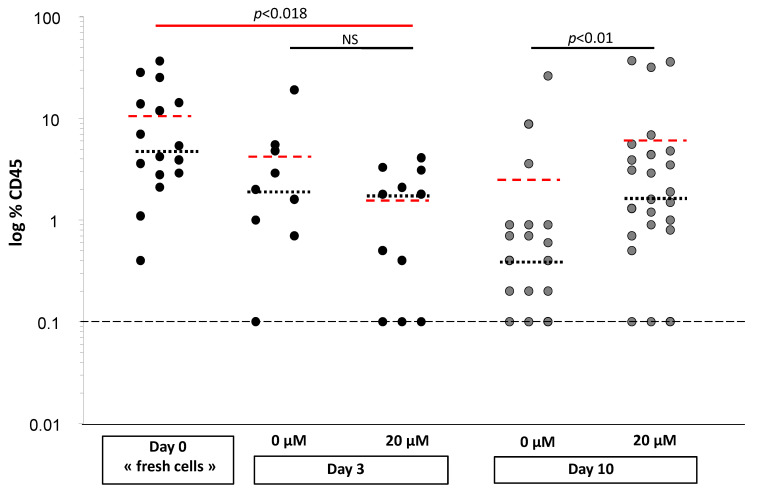
αTOA preserves a fraction of the primitive repopulating cells in culture, *n* = 3. Results were acquired in 3 independent experiments from 3 different CB units. The mice were injected with the cells on day 0 (1000 cells) and day 3 of primary culture with a cell dose representing the progeny of 1000 day 0 cells (black points: ~3 to 4 × 10^3^ cells at day 3). On day 10 of the secondary culture, a 1/10 equivalent number of day 0 cells (i.e., day 3 progeny of 1000 day 0 cells) was injected (grey points: ~64 to 82 × 10^3^ cells). In these experiments, a dose of 20 µM αTOA was applied, which was confirmed to be physiologically relevant and active. The Mann–Whitney test (black lines) showed significant results for day 10 of the secondary culture. The Kruskal–Wallis test (red line) for multiple comparisons, followed by the Mann–Whitney test with Bonferroni correction, showed significant results for day 3 with 20 µM αTOA compared to day 0. Means are represented in black dotted lines and medians in red dotted lines.

For this purpose, initially the doses of 20 and 150 µM αTOA were tested. Since the supra-physiological dose of 150 µM drastically decreased the SRCs (as shown in Supplementary Materials S1), we present only the data obtained from the 20 µM dose. The level of chimerism in mice injected with the cord blood (CB) CD34^+^ cells after 3 days of primary culture did not change significantly, as measured on the basis of human CD45 positivity (Figure 3) in the recipient bone marrow, suggesting that αTOA did not affect SRC activity. However, after 10 days of the secondary culture, a relatively higher chimerism was obtained in the group of mice injected with the cells exposed to αTOA in primary cultures. This effect can be explained either as a positive influence of αTOA on the proliferative capacity of the primitive repopulating cells that are able to generate the SRCs (pre-SRC), or as a block in the cell cycle that prevented the induction of differentiation during the initial 3 day culture and consequently resulted in better maintenance of the SRC engraftment capacity.

### 3.4. αTOA Attenuates OXPHOS in CB CD34^+^ Cells

**Figure 4:**
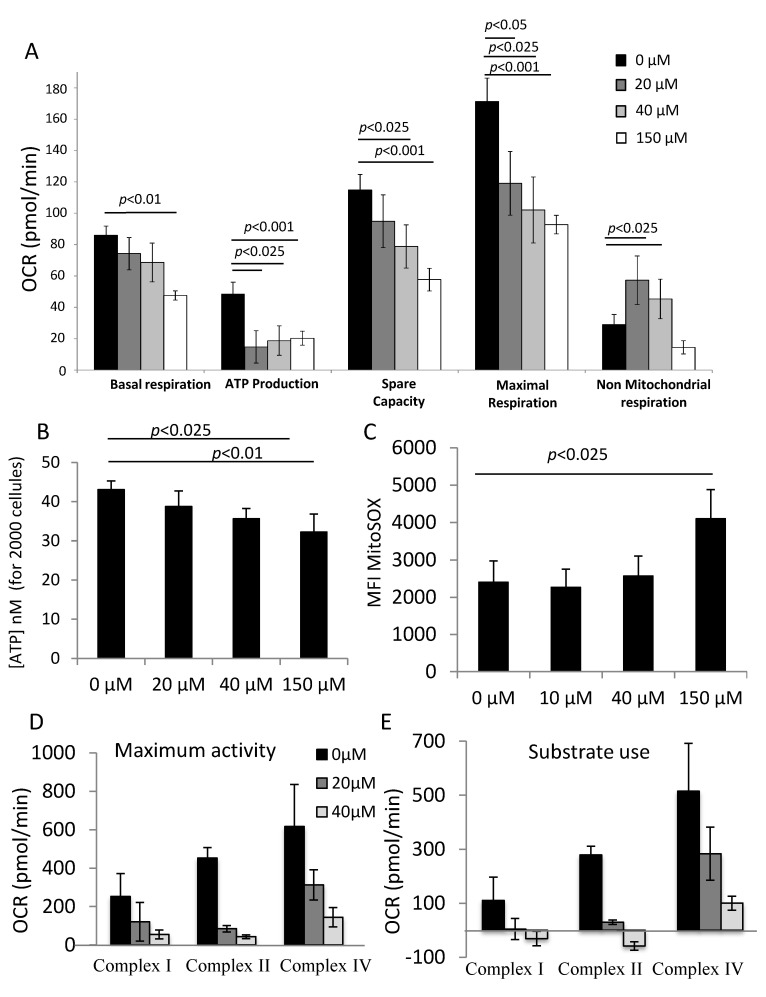
αTOA attenuates OXPHOS in CB CD34^+^ cells. (**A**) αTOA attenuates oxygen consumption rate in CB CD34^+^ cells, as well as the spare capacity and maximal capacity of OXPHOS after 24 h of culture. Seahorse indirect estimation of ATP production suggests an ATP decrease (*n* = 6), which is confirmed by direct luminometry measurement (**B**) (*n* = 6). (**C**) Mitochondrial ROS production (MitoSox probes) increases only with 150 µM of αTOA after 24 h of culture (*n* = 6). (**D**) Individual activity and substrate use (**E**) of the respiratory chain complexes I, II and IV after 24 h of culture with 20 µM or 40 µM αTOA; a decrease in activity for all complexes after αTOA application was noted (*n* = 2). Data are presented as means +/− SD. OCR = Oxygen Consumption Rate; ATP = Adenosin TriPhosphate; MFI = Mean Fluorescence Intensity.

αTOA decreased the basal respiration, spare capacity and maximal respiration rate of CD34^+^ cells in a dose-dependent manner, while the physiologically relevant doses (20 and 40 µM) significantly enhanced the non-mitochondrial respiration rate (Figure 4A). Furthermore, initial evaluation via the Seahorse parameters indicated that αTOA decreased ATP production (Figure 4A). This trend was further confirmed by direct, more sensitive and relevant luminometric ATP detection (Figure 4B).

The level of mitochondrial ROS at 24 h did not change when αTOA was added, except for the supplementation with the supra-physiological dose (150 µM). The 150 µM dose of αTOA is most likely toxic to the cells, as evidenced by the results of the apoptosis and viability assays (Figure 4C). However, for the smaller doses, a dose-dependent increase in ROS was detected after 72 h (Figure S2). The activity of the respiratory chain complexes I, II and IV was investigated only for the physiologically relevant doses (20 and 40 µM). The results indicate that at such doses, αTOA decreases the activity of complexes I, II and IV (Figure 4D,E).

### 3.5. Physiological Concentrations of αTOA Do Not Affect Glycolysis and Maintain Mitochondrial Activity

**Figure 5:**
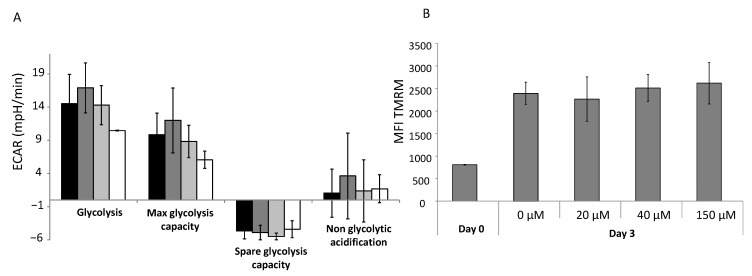
αTOA does not affect glycolysis or the mitochondrial activity of CD34^+^ cells in culture. (**A**) αTOA at physiological concentrations does not affect glycolysis (estimated on the basis of ECAR) after 3 days of culture, while the dose of 150 µM exhibits a decreasing trend of both ECAR and maximal glycolysis capacity (*n* = 6). (**B**) Global mitochondrial activity (TMRM probes) is maintained after 3 days of culture, (*n* = 3). Data are presented as means +/− SD. ECAR = Extracellular Acidification Rate; MFI = Mean Fluorescence Intensity; TMRM = TetraMethylRhodamine Methyl ester.

At physiological doses, αTOA did not affect glycolysis. This implies that glycolytic compensation for OXPHOS attenuation is lacking (Figure 5A). However, global mitochondrial activity was maintained (Figure 5B), suggesting a compensatory mechanism.

### 3.6. αTOA Does Not Interfere with the Expression of HIF-1α in CD34^+^ Cells and Inhibits the Expression of HIF-2α

**Figure 6:**
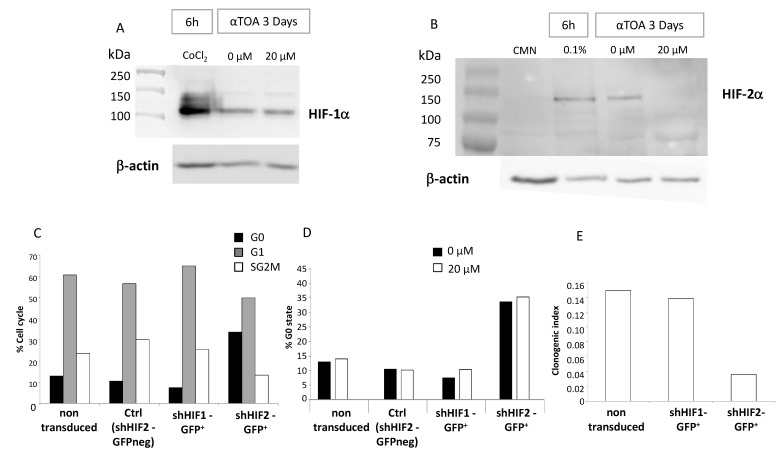
Effect of αTOA on *HIF-1α* and *HIF-2α* expression in culture. Western blot of HIF-1α (**A**) and HIF-2α (**B**) proteins extracted from CB CD34^+^ cells treated with or without αTOA during the 3 days of the primary culture. Western blots were performed twice for the detection of both HIF-1α and HIF-2α, and one representative experiment is presented. Only the 20 µM αTOA dose was tested; note the absence of the HIF-2α band in the presence of αTOA. (**C**) After 3 days of transduction with shHIF-1α and shHIF-2α, and 3 additional days of culture without αTOA, cell cycle labeling and the clonogenic index (**E**) show the effect of *HIF-2α* silencing on CB CD34^+^ cells. The 20µM αTOA concentration does not change the proportion of the G0 phase in transduced cells (mean +/− SD) (**D**). *n* = 2 for all graphs (**A**–**E**). HIF = Hypoxia Inducible Factor; GFP = Green Fluorescence Protein; sh = short hairpin; Ctrl = Control.

HIF-1α is expressed after 3 days of culture in native CB CD34^+^ cells, as well as in the cells treated with cobalt chloride (a positive control for HIF1α expression) (Figure 6A), indicating that αTOA did not interfere with *HIF-1α* expression. However, αTOA interferes with *HIF-2α* expression, as evidenced by the absence of *HIF-2α* expression in both the low O_2_ culture and in the standard oxygenation culture after the addition of 20 µM αTOA (Figure 6B). In both standard and low oxygenation cultures, *HIF-2α* expression is clearly detectable when no αTOA is present.

To test the hypothesis that the inhibition of *HIF-2α* expression by αTOA may be the induction mechanism of G0 accumulation in CD34^+^ cells, we silenced the *HIF-2α* gene in native CD34^+^ cells (a 3-day long procedure). Transduced cells (shHIF-2α-GFP^+^) without αTOA treatment underwent accumulation in the G0 phase at the expense of the G1 phase in the same way as the native αTOA-treated cells. The cells which were sorted after the transduction procedure as GFP negative (i.e., non-transduced cells) displayed the same cell cycle stage distribution as native control cells (Figure 6C). On the contrary, the effective silencing of the *HIF-1α* gene (shHIF-1α-GFP^+^) did not change the cell cycle distribution compared to the control cells (Figure 6C). The expansion of these effectively transduced cells (shHIF-2α-GFP^+^) for a further 72 h with or without αTOA resulted in further increases in the G0 fraction to the same extent, regardless of the presence of αTOA; thus, an additive effect was not evidenced. In the control cells and non-effectively transduced cells (shHIF-2α-GFP^-^), no increase in the G0 fraction was detected, irrespective of the presence of αTOA, suggesting that the effect of αTOA on G0 phase accumulation may indeed be caused by *HIF-2α* inhibition (Figure 6D). The absence of the *HIF-2α* silencing effect on the G0 phase accumulation (Figure 6D) might be explained either by the introduction of a 3-day transduction culture step or by the different levels of *HIF-2α* inhibition achieved by the silencing (transduction) and by addition of αTOA. Under the same conditions, the silencing of *HIF-1α* did not produce an effect on the G0 phase cell proportion, regardless of the presence of αTOA (Figure 6D). The *HIF-2α* inhibition-related increase in the proportion of the quiescent fraction of cells induces the inhibition of proliferation of the committed progenitors, evidenced by the decrease in the clonogenic index of shHIF-2α-GFP^+^ cells (Figure 6E), which also occurs in the same way when αTOA is added (Figure 1A).

### 3.7. In Vivo Deficiency of αTOC Promotes the Proliferation of HSPCs

**Figure 7:**
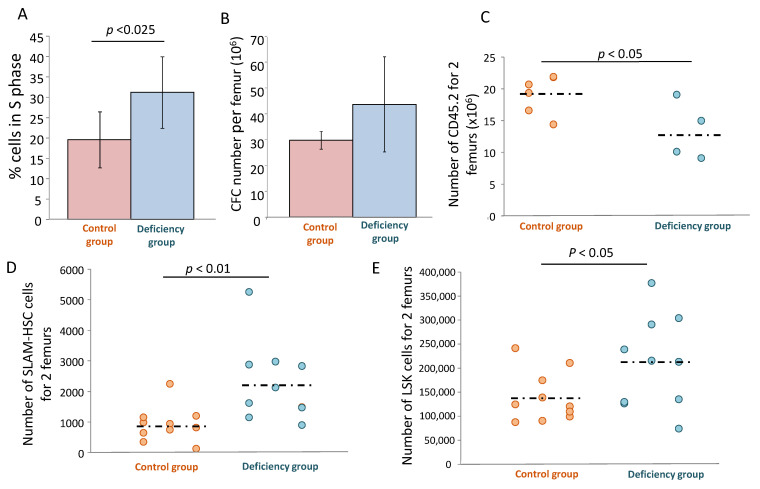
In vivo effect of αTOC on HSC and HPC. In vivo deficiency of αTOC occurring for 2 months increases the fraction of CFC in the S phase of the cell cycle (Ara-C suicide technique) i.e., it promotes the proliferation of HPCs (mean +/− SD) (**A**) and exhibits an increasing trend in the number of committed progenitors per femur (mean +/− SD) (**B**). In vivo αTOC deficiency decreases the bone marrow repopulating ability (**C**) and results in an inflation in the LSK (**D**) and SLAM cell compartments (**E**). CFC = Colony Forming Cells; HSC = Hematopoietic Stem Cells; SLAM = Cells expressing signaling lymphocytic activation molecule family receptors; LSK = Linage negative, Stem cell antigen (Sca-1) positive, C-kit positive.

Two months after inducing αTOC deficiency (following the protocol shown in Supplementary Materials S3) by the specific alimentary diet (Supplementary Materials S4), the S phase fraction of the bone marrow committed progenitors (CFU-Mix (Colony-Forming Units uf Mixed lineages), BFU-E (Burst-Forming Unit-Erythroid), CFU-GM (Colony-Forming Unit-Granulocyte Macrophage) was increased in comparison to the control mice (Figure 7A), which suggests that αTOC acts to limit the excessive cycling of these cells in physiological conditions. In addition, there is a trend of a higher amplification rate of the CFC pool in mice with αTOA deficiency (Figure 7B). The functional exploration of hematopoietic stem cells that are able to reconstitute hematopoiesis in vivo (Figure 7C) showed that αTOC deficiency actually decreased the number of HSC exhibiting a functional capacity. In spite of this fact, in terms of phenotypic populations known to be enriched in hematopoietic stem cells and primitive progenitors (respectively called SLAM (Lin^−^ CD48^−^ CD150^+^) and LSK (Lin^−^ Sca1^+^ CD117^+^) populations), αTOC deficiency resulted in their expansion (Figure 7D,E) and the consequent enhancement of the CFC compartment.

## 4. Discussion

In previous studies, it was concluded that αTOC (succinate or hemisuccinate), an αTOC derivative, does not have the same effect on normal hematopoietic cells as it does on leukemic cells [13,14]. However, to the best of our knowledge, the effect of αTOC on non-differentiated hematopoietic cells exhibiting the properties of HSPC was neither thoroughly nor rigorously investigated up until now. We investigated another αTOC analog, αTOA, which can be directly used in clinical-grade procedures. The results presented in this study show that αTOA has a toxic effect on the cells when administered in high, supra-physiological doses (150 µM), while this effect is absent at physiologically relevant doses (20 µM and 40 µM).

The ex vivo data obtained from the CB CD34^+^ cells show that αTOA exhibits multiple effects on HSPCs, leading to increases in the HSPC proportion “arrested” in the quiescent state (G0 phase), as well as improved maintenance of their proliferative capacity. This latter effect may be relevant for HSC and HPC ex vivo engineering, particularly for the protocols where expansion should be combined with a “purge” that targets actively cycling cells. Alternatively, this approach might be used in ex vivo protocols for the amplification of the HPC fraction to prevent exhaustion of HSCs. We show that αTOA attenuates mitochondrial OXPHOS; however, the main mechanism underlying the accumulation in the G0 phase appears to be related to the inhibition of HIF-2α expression by αTOA. Although more thorough research is necessary to reveal the exact link between the attenuation of OXPHOS and *HIF-2α* expression, an increase in ROS (detected after 72 h in our case) cannot be discarded. The increase in ROS is expected when real attenuation of OXPHOS occurs, such as when applied HIF-2α causes a decrease in ATP levels in a dose-dependent manner. Our results point to a blockage of the respiratory chain complexes I and II, which prompted us to hypothesize that αTOA may compete for electrons with ubiquinone/ubiquinol (coenzyme Q10 (CoQ10)), a molecule of very similar structure [15,16] that is critical for electron transport activity. αTOA may, in competition with CoQ10, undergo cyclic oxidation–reduction during the oxidation of substrates in the citric acid cycle, i.e., the reduction of fumarate to succinate. This hypothesis remains to be further explored, as well as the one concerning the effect of αTOA on the metabolism of lipids and amino acids in order to explain the sustained mitochondrial activity (ongoing work).

It is interesting to note that another αTOC supplement, α-tocopheryl succinate (αTOS), induces apoptosis in cancerous and leukemic cells via the blockage of either complex I [17] or complex II activity by interacting with the proximal and distal ubiquinone-binding sites [18].

Based on the data presented in this study, we conclude that αTOA does not affect CD34^+^ cells in the active phases of the cell cycle, but rather prevents their exit from the G0 phase. When applied at the non-physiological, toxic dose of 150 µM, αTOA caused re-entrance of the cells into the G0 phase. This effect was shown for CB CD34^+^ cells stimulated by IL-3 in response to an extremely low O_2_ concentration [11]; however, in the case of αTOA, it cannot be regarded as relevant due to the toxicity of the applied dose. It is possible that the higher proliferative capacity of the cells cultured in the presence of αTOA, revealed by an enhanced repopulation of the secondary cultures by HPC (CFC) and HSC (SRC), simply results from a better preserved quiescent fraction of the HSC. This is why the “HIF-2α issue” emerges as the most interesting point of this study. Treatment with αTOA abrogates HIF-2α expression (starting from the RNA level (Figure S3)) and induces an accumulation of CD34^+^ cells in the G0 phase. This G0 accumulation is also induced by shHIF2-α treatment, but it is not enhanced if αTOA is added simultaneously with shHIF-2α. The absence of an additive or synergic effect suggests that both shHIF2-α and αTOA target the same mechanism. The data presented here clearly show that the silencing of HIF-2α synthesis leads to an increase in the G0 proportion of human CD34^+^ cells and, simultaneously, to the better maintenance of primitive HSC proliferative capacity/clonogenic potential. However, one cannot claim that HIF-2α is not a positive factor in HSC maintenance on the basis of these data obtained with CD34^+^ cells. In fact, real HSC are a minority inside the CD34^+^ cell population. Thus, we cannot question the result of the previous study with human cells [19]. The results in mice showed that the positive in vivo effect of HIF-2α on the maintenance of hematopoiesis is associated with the functional bone marrow microenvironment [20], while HIF-2α is not relevant for the maintenance of the HSCs themselves [21]. Hence, in regard to this observation, it has been considered that HIF-2α regulatory competencies may be different in human and in mouse [22]. The cellular and molecular responses depend on a cocktail of cytokines and other conditions of culture, so a more thorough investigation is necessary to understand the HIF-2α regulatory network.

The inhibitory effect of αTOA on HIF-2α can be realized via Phosphatidylinositol-3-Kinase and Protein Kinase B (Pi3K/Akt) signaling [22], a key mechanism related to the proliferative fate of cells. Enhanced Pi3k/Akt pathway activation results in HIF-2α overexpression [23], so one might suppose that the opposite action also operates, which is a hypothesis that remains to be explored. In addition, our proteomic analysis showed a downregulation of the Pi3K signaling pathway protein in CB CD34^+^ cells after 3 days of culture with αTOA (data not shown).

Moreover, the inhibition of cyclin D1 by αTOA in CB CD34^+^ cells could also be related to the Pi3K/Akt pathway [24]. It has already been shown that γ-tocopherol inhibits cell cycle progression and cyclin D1 expression in human cancer cells [25]. In our study, we show that αTOA can also modulate the cell cycle by decreasing cyclin D1 expression, most likely via the downregulation of the Pi3k/Akt pathway, which might explain the final effect of αTOA on the G0 accumulation of CD34^+^ cells that we detected.

As a matter of fact, the culture that was not supplemented with αTOA should be considered rather as a vitamin E deficiency model [3]. Indeed, our proteomic analysis (data not shown) confirms a high activity of adipocyte plasma membrane associated protein (APMAP), exhibiting a high arylesterase activity, as well as seven enzymes of the mevalonate pathway (whose precursor is acetate). Thus, as in other cell types [2,26], it is apparent that in CD34^+^ cells, αTOA is hydrolyzed. In this view, αTOA supplementation yields αTOC, thus resolving αTOC deficiency and enabling the creation of a biomimetic environment as a better analogue of the physiological one. In this respect, one can hypothesize that one of the physiological roles of αTOA is related to HSC maintenance. The in vivo data presented here seem to be in favor of this hypothesis, since we found the signs of partial exhaustion of the primitive HSCs (in vivo repopulating cells), both in terms of their functional activity (in vivo repopulation test) as well as in terms of inflation of the phenotypically-defined bone marrow SLAM and SLK populations, which was not accompanied by an increase in engraftment capacity. One may further hypothesize that αTOC might protect the HSC from cycle-specific agents in vivo, or even from ionizing radiation, which remains to be explored. The enhanced compartment of SLAM and LSK (Figure 7D,E) can be explained by the well-known phenomenon of phenotype/function dissociation [27,28]. Furthermore, we found an enhanced proliferative activity at the level of the committed progenitors in mice with αTOC deficiency (which is opposite to the ex vivo inhibition of proliferation by αTOA). Therefore, without αTOC, HSC and HPC accelerate their proliferation and lose their proliferative capacity. It should be stressed that in these experiments, we provoked only a relative deficiency (Figure S4) of αTOC. Thus, the effects demonstrated here would most likely be even more pronounced in a real model of αTOC deficiency. If we consider these results bearing in mind the historical data concerning the clinical cases of αTOC deficiency (both in human and veterinary medicine), we can recognize an excessive proliferation of committed progenitors and precursor cells in view of an erythroid hyperplasia with multinucleated erythroid precursors in rhesus monkeys [29] and rabbits [30]. Other reports consider the hemolytic anemias in preterm human neonates [31,32] and in owl monkeys [33]. Another interesting detail that we found is the phenomenon of the heart-shaped BFU-E colonies grown from the cells that were cultured in the presence of αTOA (Figure S5). This phenomenon might reveal an effect on the asymmetrical proliferation of descending populations (CFU-E and precursors), or an influence of αTOA on the migration of progenitors or/and precursors, which all remains to be explored in more detail.

The improved maintenance of HSC was seen concerning the effects of αTOA on MSCs [3] whose mitochondrial respiration was impacted, similarly to the results of our study. However, all other effects were different in comparison to those evidenced in CD34^+^ cells, i.e., αTOA did not impact MSC cycling and did not have an effect on HIF-2, but it did stabilize HIF-1α, which seems to be related to the ROS increase detected at 24 h after αTOA treatment. Thus, the biochemical and molecular response of αTOA appears to be dependent on the cell type. However, similarities in the results for MSC and HSC seem to indicate a positive effect of αTOA on the maintenance of stem cells.

## 5. Conclusions

α-Tocopherol attenuates the oxidative phosphorylation of CD34^+^ cells, enhances their G0 phase fraction and promotes hematopoietic stem and primitive progenitor cell maintenance. Since αTOA affects the proliferative capacity of HSCs, its use in HSC engineering is possible. Furthermore, αTOA is an injectable pharmaceutical product that enables a direct translation to clinical use. Importantly, αTOA should be applied at a physiological dose only, since higher concentrations can have a toxic effect. Taken together, the results of both ex vivo and in vivo experiments described in our study suggest a physiological role for αTOC in HSPC maintenance.

## Data Availability

All data are available at the internal server of EFS-NVAQ Bordeaux upon request.

## Supplementary Materials

The following are available online at https://www.mdpi.com/article/10.3390/biom11040558/s1, Figure S1: αTOA at a supra-physiological dose (150 µM) decreases SRC activity after 3 days of culture, Figure S2: Mitochondrial ROS increase after 3 days of primary culture with αTOA, *n* = 3, Figure S3: Experimental protocol for the in vivo determination of αTOC action, Figure S4: Levels of αTOC in mice sera after 2 months of specific diet consumption, Figure S5: Heart-shaped BFU-E colonies in the methylcellulose cultures grown from cells treated with αTOA. In addition to Figures S1–S5, the complete blots for HIF-1α and HIF-2α are presented.
