# Supplementary material for: α-Tocopherol Attenuates Oxidative Phosphorylation of CD34+ Cells, Enhances Their G0 Phase Fraction and Promotes Hematopoietic Stem and Primitive Progenitor Cell Maintenance
Source: Biomolecules. 2021 Apr 10;11(4):558. doi: 10.3390/biom11040558 (PMC8070309; doi:10.3390/biom11040558)
Supplement: Supplementary file 1 [file biomolecules-11-00558-s001.pdf]

Supplementary Materials

S1

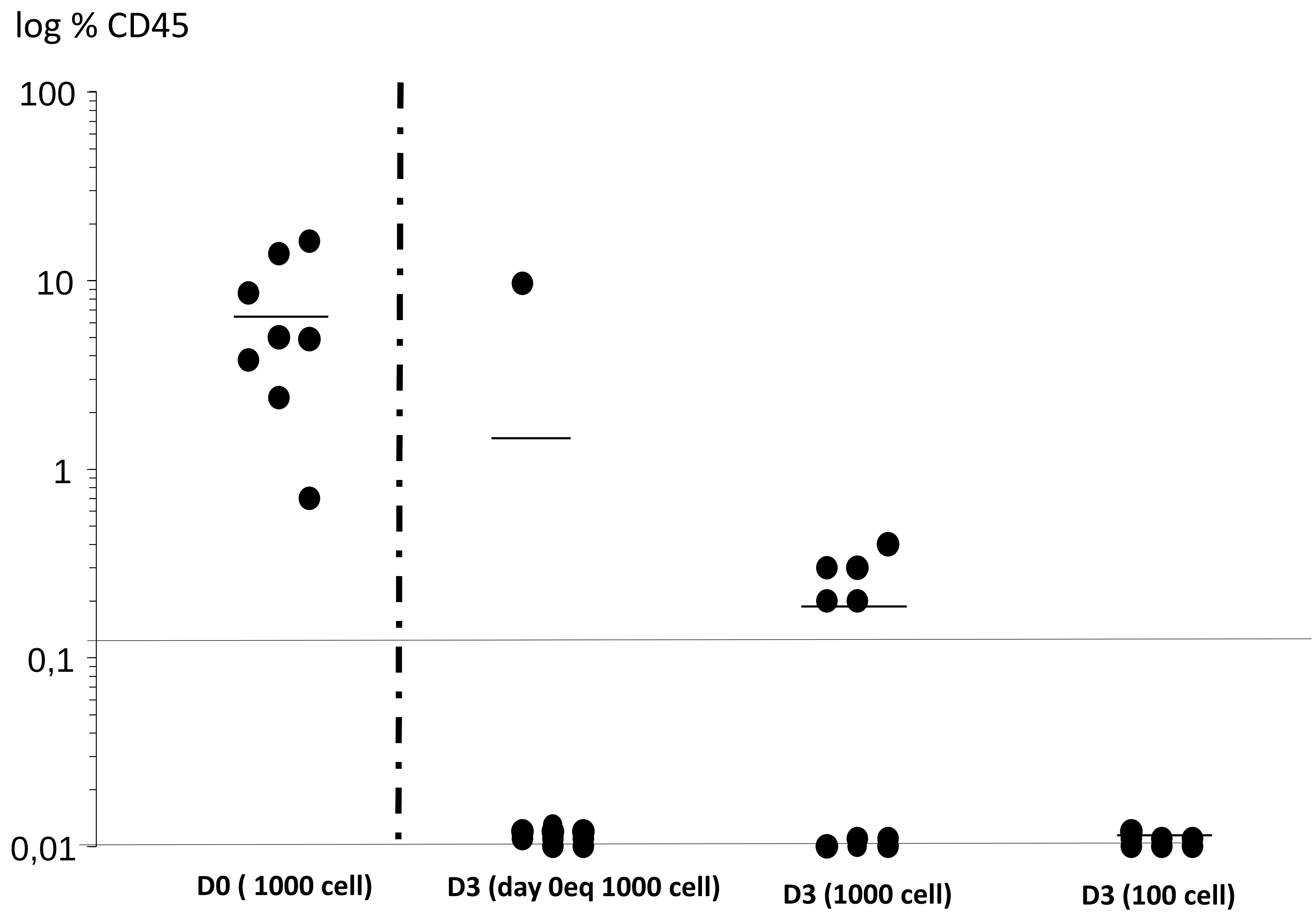

αTOA in supra-physiological dose (150µM) decreases SRC activity after 3 days of culture.

S2

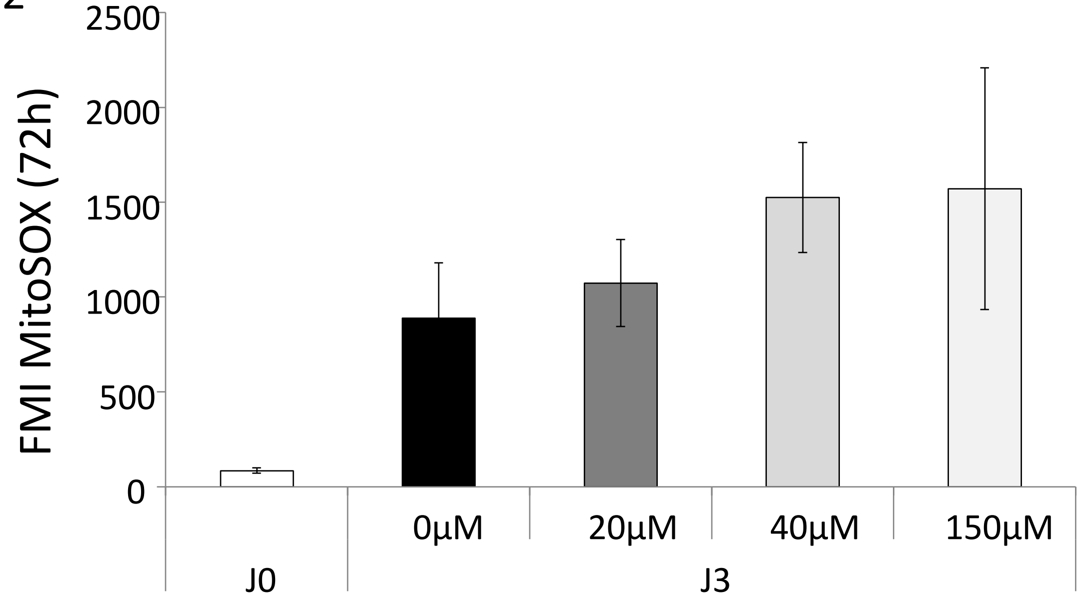

Mitochondrial ROS increase after 3 days of primary culture with αTOA. N = 3

S3

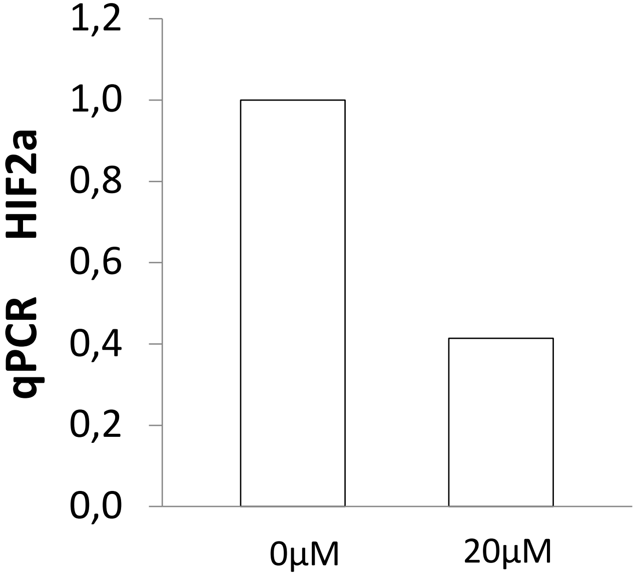

mRNA of HIF2α decrease after 3 days with 20 µM of αTOA. N = 2

S3

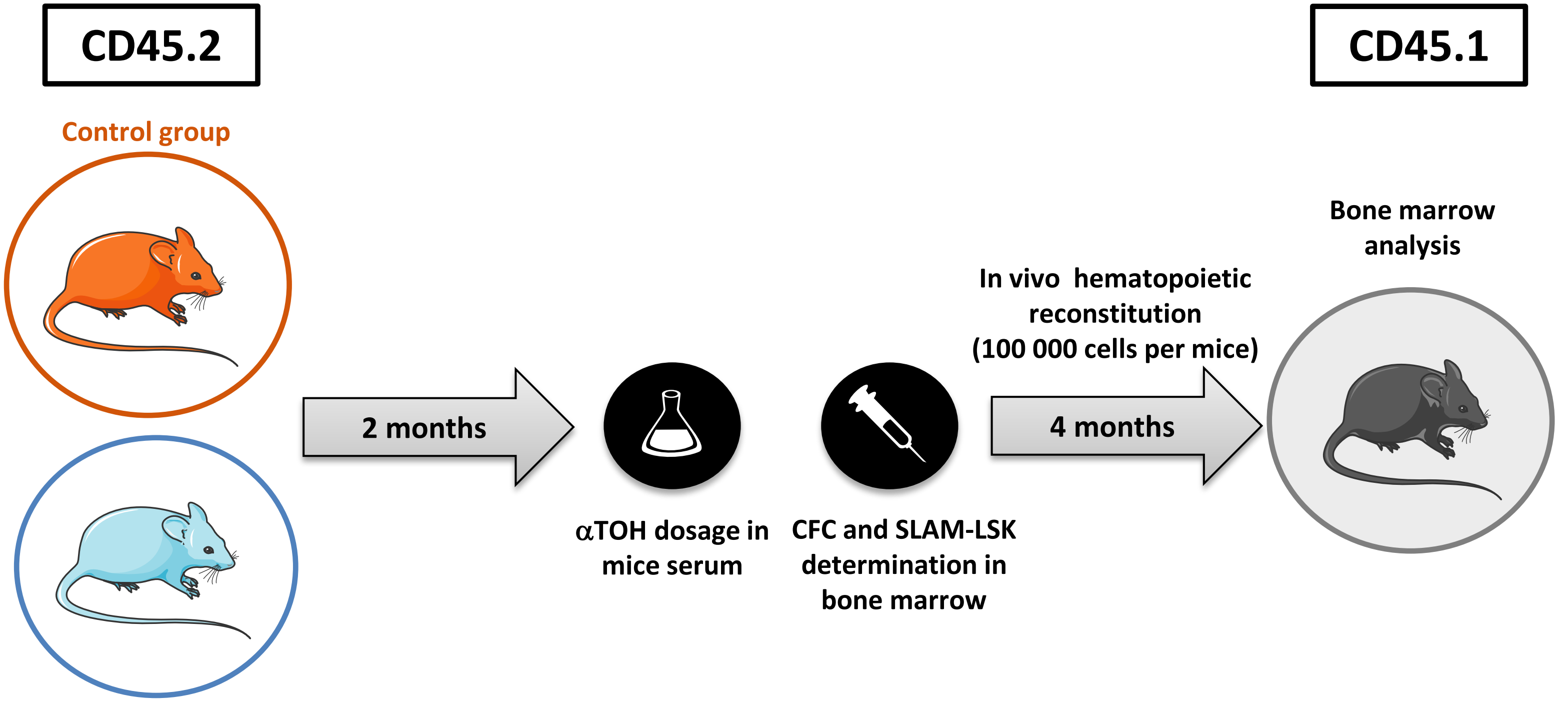

Experimental protocol for *in vivo* determination of αTOC action

S4

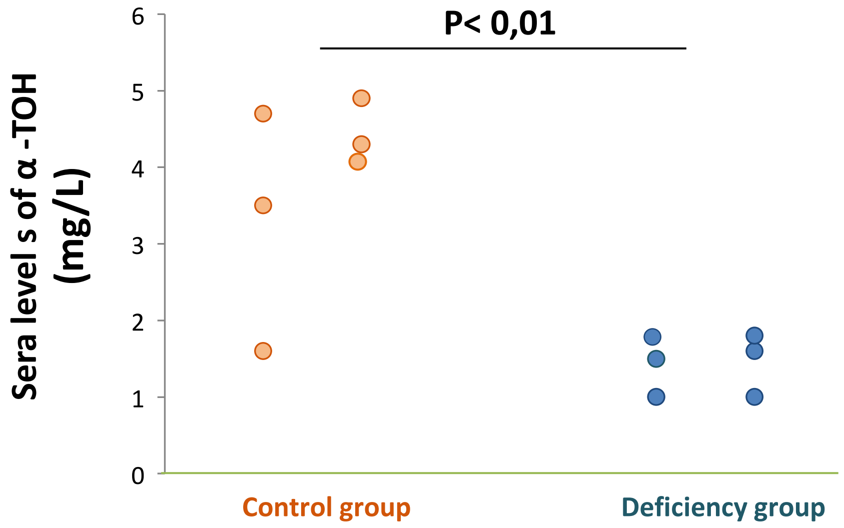

Levels of αTOC in mice sera after 2 months of specific diet

S5 heart-shaped BFU-E colonies

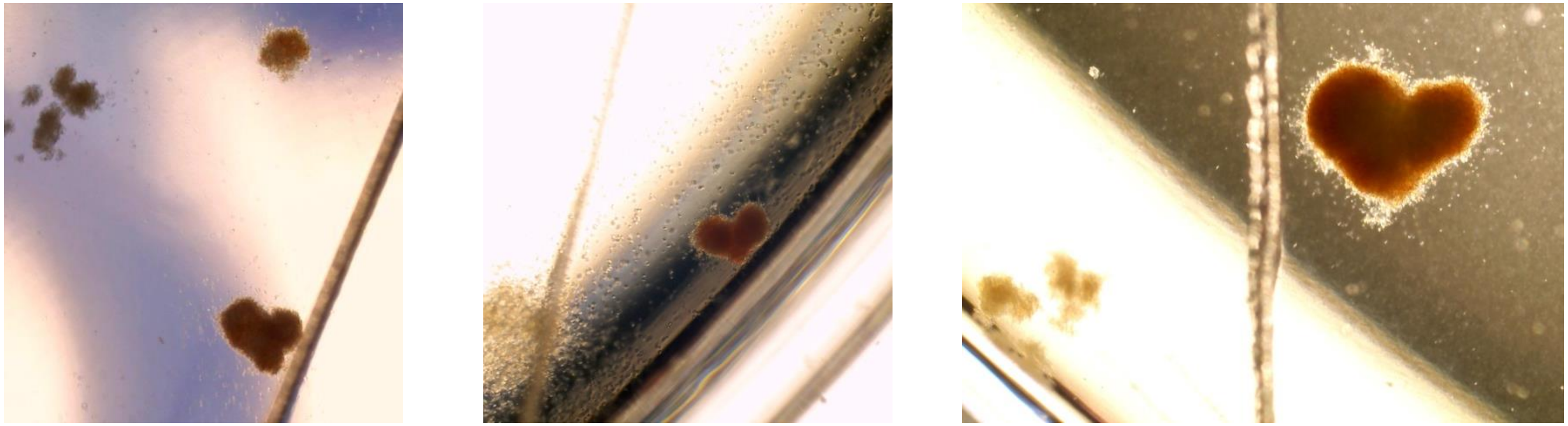

Heart-shaped BFU-E colonies in the methylcellulose cultures grown from cells treated with αTOA

Complete Blots of HIF2α

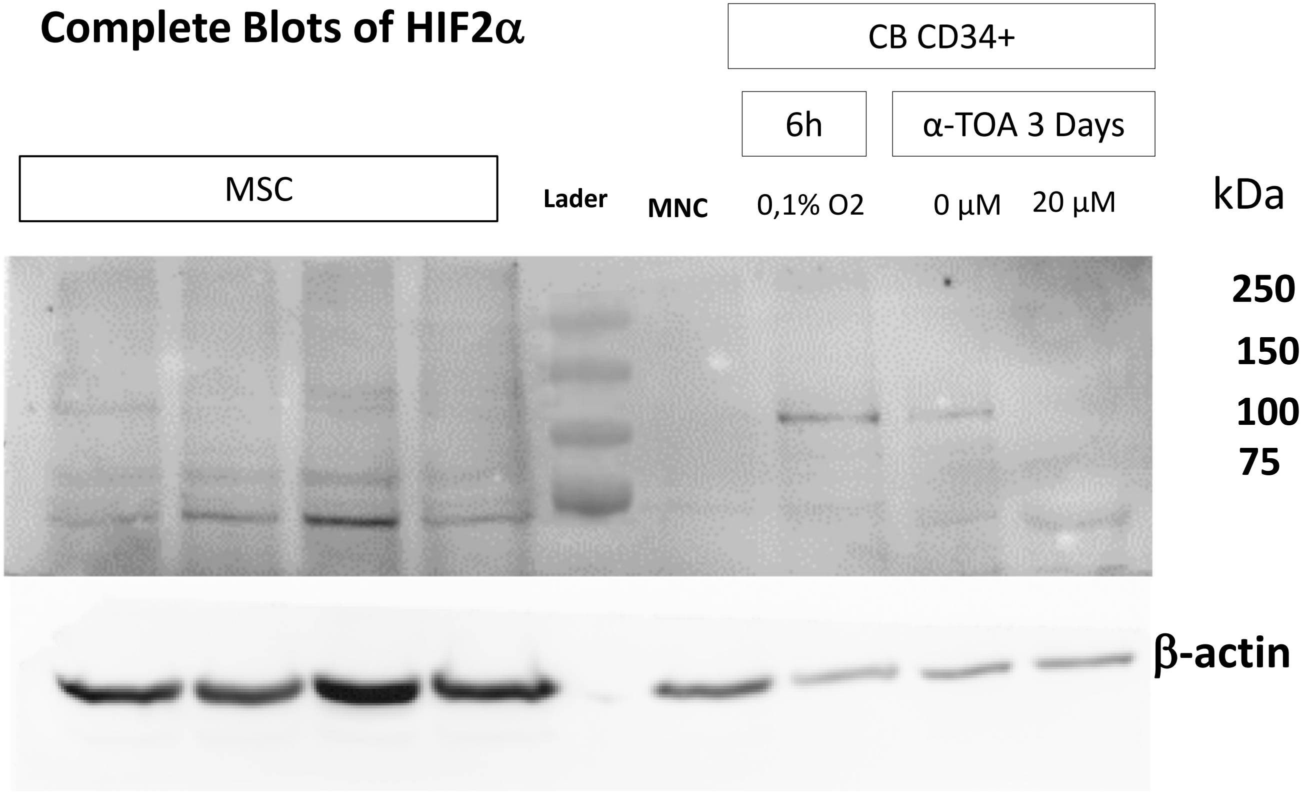

Complete Blots of HIF1α

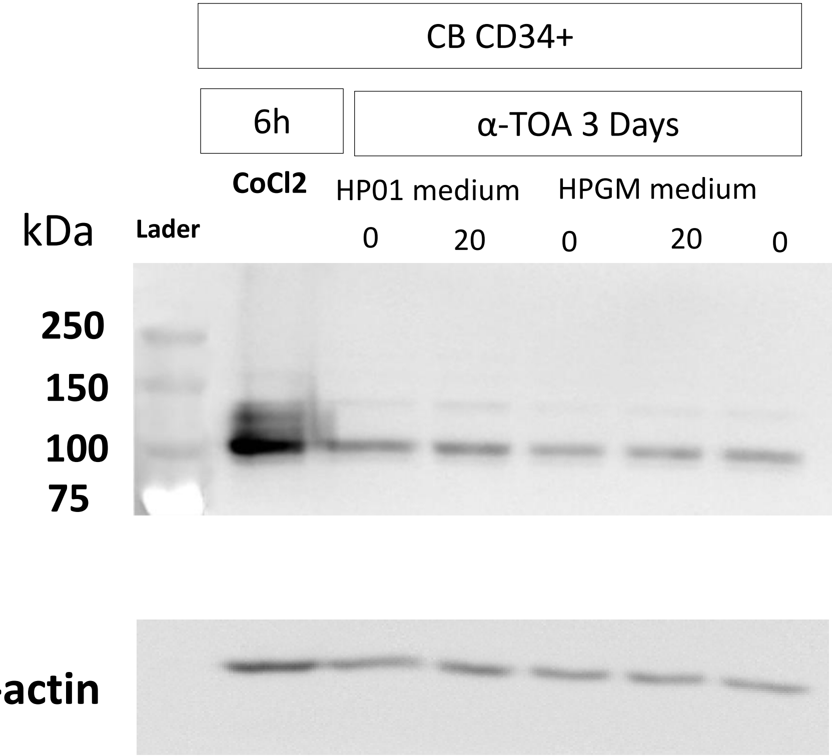

- In order to reveal primary antibody (anti-HIFs) and loading control in the same time we cut the membrane in two parts.
- HIFs proteins have a higher molecular weight (<75kDa) and b-actin a smaller one (43kDa) and all bands are in line according to predicted size.
- Left part of HIF2α blot was removed for Figure 6 because tested samples were from an other human cell type (Mesenchymal Stromal Cells - MSC)
- Right part of HIF1α blot was removed for Figure 6. We tested an other culture medium.
